# Cadmium Stress Leads to Rapid Increase in RNA Oxidative Modifications in Soybean Seedlings

**DOI:** 10.3389/fpls.2017.02219

**Published:** 2018-01-09

**Authors:** Jagna Chmielowska-Bąk, Karolina Izbiańska, Anna Ekner-Grzyb, Melike Bayar, Joanna Deckert

**Affiliations:** ^1^Department of Plant Ecophysiology, Faculty of Biology, Institute of Experimental Biology, Adam Mickiewicz University in Poznań, Poznań, Poland; ^2^Department of Molecular Biology and Genetics, Faculty of Science, Istanbul University, Istanbul, Turkey

**Keywords:** cadmium, soybean, RNA oxidation, 8-hydroxyguanosine, AP-sites, abasic sites, epitranscriptomics, oxidative stress

## Abstract

Increase in the level of reactive oxygen species (ROS) is a common response to stress factors, including exposure to metals. ROS over-production is associated with oxidation of lipids, proteins, and nucleic acids. It is suggested that the products of oxidation are not solely the markers of oxidative stress but also signaling elements. For instance, it has been shown in animal models that mRNA oxidation is a selective process engaged in post-transcriptional regulation of genes expression and that it is associated with the development of symptoms of several neurodegenerative disorders. In the present study, we examined the impact of short-term cadmium (Cd) stress on the level of two RNA oxidation markers: 8-hydroxyguanosine (8-OHG) and apurinic/apyrimidinic sites (AP-sites, abasic sites). In the case of 8-OHG, a significant increase was observed after 3 h of exposure to moderate Cd concentration (10 mg/l). In turn, high level of AP-sites, accompanied by strong ROS accumulation and lipid peroxidation, was noted only after 24 h of treatment with higher Cd concentration (25 mg/l). This is the first report showing induction of RNA oxidations in plants response to stress factors. The possible signaling and gene regulatory role of oxidatively modified transcripts is discussed.

## Introduction

Elevated levels of cadmium (Cd) were found in the soil in several regions of the world (Khan et al., 2011; Wang et al., 2015; Dumitrel et al., 2017). Contamination of the environment with this metal possesses serious threat to plants growth as Cd is highly mobile and toxic. Importantly, it can be absorbed by crop plants and in this mode enter human organisms leading to serious disorders. Cd is considered as class I carcinogen. The main targets of its toxicity are kidneys, lungs, and skeleton system (Kah et al., 2012).

In recent years, significant effort has been made to elucidate Cd impact on plants. The research has been focused on the toxicity mechanisms, Cd sensing, and the development of metal tolerance. The most universal responses to this toxic element, not only limited to plants but also found in bacteria and animals, are over-production of ROS (reviewed in Chmielowska-BąK and Deckert, 2012). ROS are “double-faced” molecules, which on one hand can lead to significant damage of cellular compounds but on the other hand are crucial components of signaling network indispensable for the activation of the defense (Cuypers et al., 2016; Sewelam et al., 2016). Recently it has been proposed that ROS signal is transmitted by the products of the oxidation of biological molecules including lipids, proteins, and nucleic acids (reviewed in Chmielowska-BąK et al., 2015).

Indeed, various studies carried out on human, animal, and plant system demonstrated correlation between oxidation of certain species of mRNA and decrease in the level of encoded proteins, indicating that the process constitutes a post-transcriptional gene regulatory mechanism (Shan et al., 2003, 2007; Tanaka et al., 2007; Chang et al., 2008; Bazin et al., 2011; Gao et al., 2013). The decrease in the amount of proteins encoded by oxidized transcripts is most probably dependent on recently described ribosome stalling. *In vitro* studies carried out on reconstituted bacterial system demonstrated that occurrence of 8-OHG, the most common oxidative modification of RNA, causes slowing down of translation process by 2–4 magnitudes. The effect has been observed regardless of the position of oxidized bases in the codon, even in the wobble position. In eukaryotic extracts, translation was nearly completely inhibited by the presence of 8-OHG. It was suggested that occurrence of 8-OHG in transcripts leads to alterations in RNA–RNA interactions and prevents adaptation of active conformation in the decoding center. At the same time, it has been shown that oxidized transcripts are subjected to ribosome-based quality control and are predestined for degradation through No-Go decay pathway (NGD) (Simms et al., 2014). In concordance, yeast mutants with alerted decapping system leading to less efficient mRNA degradation showed elevated level of 8-OHG in the transcripts accompanied by premature death of the cells. The yeast mutants were also characterized by higher frequency of reversion from Trp^-^ (tryptophan minus) phenotype (Stirpe et al., 2016).

Another major discovery is the fact that mRNA oxidation is a selective process. Study carried out on postmortem isolated brain tissues of patients suffering from Alzheimer’s disease demonstrated that oxidation did not occur in the most abundant transcripts such as β*-actin*. On the other hand, the most prominent oxidation was always noted in specific mRNAs encoding proteins engaged in signal transduction, cellular transport, gene expression regulation, and response to alerted ROS metabolism (Shan et al., 2003). These findings have been confirmed by other reports carried out on human, animal, and plant models (Shan et al., 2007; Tanaka et al., 2007; Chang et al., 2008; Bazin et al., 2011; Gao et al., 2013). Despite the importance of transcript oxidation in genes regulation and cell functioning so far, only two studies were dedicated to elucidation of this phenomenon in plants. It was demonstrated on sunflower seeds that alleviation of seed dormancy during dry after-ripening was associated with increase in the 8-OHG in mRNA. The observed oxidation was limited to 24 definite transcripts encoding proteins associated with metabolism, response to stress factors, and transport (Bazin et al., 2011). Similarly, studies on wheat showed selective oxidation of certain transcripts associated with changes in protein levels and release from seed dormancy (Gao et al., 2013).

Beside the 8-OHG, oxidation might lead to the formation of numerous other modified bases in nucleic acids (Barciszewski et al., 1999). Studies using *in vitro* translation system showed that the most common oxidative modifications of RNA, namely 8-OHG, 5-hydroxyuridine (5-OHU), 5-hydroxycytidine (5-OHC), 8-oxo-7,8-dihydroadenosine (8-OHA), 1,N6-ethenoadenosine (ε-A), 3,N4-ethenocytidine (ε-C), and abasic sites (AP), result in slowing down or complete inhibition of the translation process (Calabretta et al., 2015). In turn, oxidation of DNA is associated with decrease in its stability and enhanced mutation rate. High level of oxidatively modified bases has been noted in various types of cancer (Sedelnikova et al., 2010). The repair of DNA lesions induced by oxidation is carried out by the Base Excision Repair (BER) pathway. The initial step of BER is recognition and excision of the modified bases by DNA glycosylases leading to the formation of AP-sites (abasic sites), which are considered markers of oxidative stress (Čolak, 2008; Antoniali et al., 2017). Recently, a method of abasic sites detection has been successfully applied in the evaluation of RNA oxidation (Tanaka et al., 2011). However, so far the changes in the level AP-sites in transcripts of plants exposed to stress factors have not been described.

The aim of present study was examination of the influence of Cd in two concentrations (10 and 25 mgl^-1^) on the intensity of oxidative stress and frequency of RNA oxidation-dependent modifications – 8-OHG and AP-sites. Our previous research showed that in the earliest period of Cd stress (3 h), ROS are engaged in the regulation of gene expression, while strong accumulation of O_2_^-^ and H_2_O_2_ has been marked after 24 h (Chmielowska-BąK et al., 2017). Therefore, these two time points, 3 and 24 h, were applied in the present study.

## Materials and Methods

### Growth Conditions and Treatment Procedures

Soybean (*Glycine max* L cv. Naviko) seeds were kindly supplied by the Department of Genetics and Plant Breeding, University of Life Sciences in Poznań, Poland. The seeds were surface-sterilized for 5 min with 75% ethanol and for 10 min with 1% sodium hyperchlorite. Thereafter, the seed were washed under running water for 30 min and imbedded in distilled water for 2 h. The seeds were placed on Petri dishes (30 cm of diameter) lined with two layers of moistened lignin covered by one layer of blotting paper and transferred to growth chamber with stable temperature of 22°C for 48 h. Germinated seedlings, selected in respect of similar roots length, were transferred to new Petri dishes (10 cm of diameter), wherein the roots were placed between two layers of blotting paper in cutout wholes. Afterward, the seedlings were treated with 5 mL of distilled water (control) or CdCl_2_ with Cd at the concentration 10 and 25 mg l^-1^ (corresponding to 89 and 223 μM, respectively).

### Estimation of the Amount of Dead Cells

Cell viability was estimated on the basis of Blue Evans uptake according to Lehotai et al. (2011). Approximately 200 mg of roots were cut off on ice, weighted (200 mg), and incubated for 20 min in 0.25% Evans blue (Sigma, E-2129). Then the roots were washed twice for 15 min in distilled water and homogenized in mortar with – destaining solution (50 ml of ethanol, 49 ml of distilled water, and 1 ml of 10% SDS). Samples were incubated in heating block for 15 min at 50°C and centrifuged (12,000 rpm, 20°C, 15 min). The Blue Evans uptake, indicating cells death, was measured spectrophotometrically at λ = 600 nm.

### Total RNA and mRNA Isolation

For RNA isolation, approximately 100 mg of soybean roots were cut off on ice, immediately frozen in liquid nitrogen, and stored in -80°C. The RNA was isolated from frozen tissue with the use of TriReagent (BioShop Canada Inc., Canada, TRI118) according to the manufacturer’s instructions. The mRNA has been purified from the total RNA using GenElute^TM^ mRNA Miniprep Kit (Sigma–Aldrich, MRN10-1KT). The amount and purity of the obtained RNA and mRNA has been measured on NanoCell (Thermo Scientific) at spectrophotometer Biomate^TM^ 3S (Thermo Scientific).

### Measurement of 8-OHG Level

The level of 8-OHG has been quantified with OxiSelect^TM^ Oxidative RNA Damage ELISA-8OHG Quantification Kit (BioCells, STA-325). For the analysis, 10 μg of sample (total RNA or mRNA) was digested with 20U of Nuclease S1 (BioShop Canada Inc., Canada, NUC333.50) for 2 h at 37°C followed by digestion with 10U of alkaline phosphatase from bovine intestinal mucosa (Sigma–Aldrich, P6774-2KU) for 1 h at 37°C. Further procedures were carried out according to the manufacturer’s instructions. The absorbance of the samples was measured on İMARK^TM^ Microplate Reader (Bio-Rad) and the 8-OHG concentrations were calculated using ELISA Analysis software with 4-parameter logistic regression algorithm.

### ROS Detection

The general ROS were detected *in vivo* in the roots of soybean seedlings using fluorescent dye, CM-H_2_DCFDA (Life Technologies, C6827) dissolved in dimethyl sulfoxide (DMSO; Sigma–Aldrich, 472301) and diluted in phosphate-buffered saline (PBS) buffer (BioShop Canada Inc., Canada, PBS404) to the total concentration of 10 μM. The roots of seedlings were incubated for 1 h in CM-H_2_DCFDA, washed with distilled water, and treated for 3 or 24 h with Cd solutions (10 or 25 mg L^-1^) or distilled water (experimental control). To exclude the possibility of autofluorescence, the negative control incubated for 1 h in PBS buffer instead of CM-H_2_DCFDA has been applied. All procedures were carried out in the dark room. The level of general ROS was visualized by means of Zeiss Axiovert 200M confocal microscope with 450–490 nm excitation and 515 nm emission light wave length. The 5× magnified images were photographed using AxioCam MRC5 camera.

### Measurements of Lipid Peroxidation

Lipid peroxidation was evaluated on the basis of the amount of TBARS according to Cuypers et al. (2011) with small modifications. Roots of soybean seedlings (200 mg) were cut off on ice and homogenized with 3 ml of 10% TCA (Sigma–Aldrich, TO699). The samples were centrifuged (12,000 rpm, 4°C, 10 min) and 1 ml of supernatant was transferred to glass tubes. Thereafter, the tubes were filled with 4 ml of 0.5% TBA, dissolved in 10% TCA, and incubated for 30 min in 95°C. Subsequently, the samples were cooled, mixed by inversion, and centrifuged (5,000 rpm, 4°C, 2 min). The absorbance of supernatant was measured at λ = 532 nm and corrected for unspecific absorbance at λ = 600 nm. Amount of TBARS was calculated on the basis of extinction factor (155 mM^-1^cm^-1^).

### Estimation of the Level of AP-Sites in RNA

The level of abasic sites (AP-sites) has been evaluated using method based on reaction with ARP described by Tanaka et al. (2011). Approximately 5 μg of mRNA dissolved in 50 μl of DNAase- and RNAase-free water was incubated with 50 μl of 2mM N-(aminooxyacetyl)-N′-(D-Biotinoyl)hydrazine (Life Technologies, A-10550) in Tris–EDTA buffer (Sigma–Aldrich, T9285) for 1 h at 37°C. The reaction was stopped by addition of 50 μl of 50 mM formaldehyde (BioShop Canada Inc., Canada, FOR201.1). RNA precipitation has been carried out by addition of 15 μl of 3M sodium acetate (BioShop Canada Inc., Canada, SAA333.100) and 450 μl of pure ethanol (POCH Basic, BA 6480111). The precipitation proceeded for 24 h at -20°C. Thereafter, the samples were centrifuged by 12,000 rcf at 4°C, washed with 500 μl of 75% ethanol and dissolved in 10 μl of Tris–EDTA.

The amount and purity of the obtained mRNA has been measured on NanoCell (Thermo Scientific) at spectrophotometer Biomate^TM^ 3S (Thermo Scientific). A total of 1 μg of the mRNA has been spotted on membrane (Zeta-Probe^®^ Blotting Membranes, Bio-Rad) previously soaked in Tris–EDTA and air-dried. The membrane with samples was irradiated for 15 min with UV light, incubated for 30 min in Casein Blocking Buffer (Sigma–Aldrich, B6429), followed by incubation for 1 h with Streptavidin-Horseradish Peroxidase (HRP) Conjugate (Sigma–Aldrich, GERPN1231) in Casein Blocking Buffer (1:20,000). Thereafter, the membrane was washed 6 times for 4 min with PBS (BioShop Canada Inc., Canada, PBS404.200) containing 0.05% Tween (Sigma–Aldrich, P1379) and developed with Clarity^TM^ Western ECL Substrate (Bio-Rad). The blocking, incubation with streptavidin-HRP, washing, and developing proceeded on rocking platform (Shaker-Rocker MR12, BioSan). Chemiluminescence has been captured on ChemiDoc^TM^ Touch Imaging System (Bio-Rad) with 15 min exposure time. The intensity of spots was measured with Multi Gauge Software (Fuji) as Q-B/pixel^2^, where Q is quantity and B is background. The relative density has been expressed as percentage in relation to the control.

### Statistical Analysis

The measurements of the 8-OHG level in total RNA and mRNA were carried out on 4 and 5–6 experimental repetitions, respectively. The measurements of growth, lipid peroxidation, and abasic sites level in mRNA were conducted on 3 experimental repetitions, while the estimation of the amount of dead cells in 2–3 experimental repetitions. For evaluation of statistically significant differences, obtained data were analyzed with the use of one-way ANOVA (*p* = 0.05). In the case of evaluation of the level of abasic sites in mRNA, due to the non-parametric distribution, the Mann–Whitney *U*-test has been applied (*p* = 0.05). Results which showed no statistically significant differences are marked with the same letter.

## Results

**FIGURE 1:**
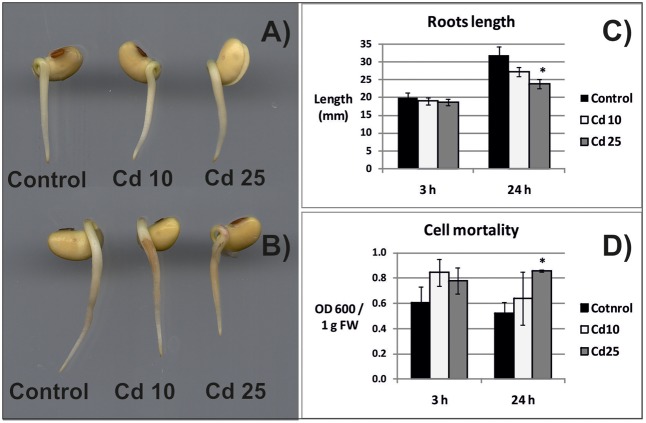
Morphology of the control seedlings and seedlings treated with Cd for 3 h **(A)** and 24 h **(B)**, length of seedlings roots **(C)**, and cell mortality evaluated on the basis of Blue Evans uptake **(D)**. The results are means of 3–6 independent repetitions ± SE. Results marked with asterisk (^∗^) show statistically significant differences in relation to the control.

Exposure of seedlings to Cd for 3 h had no effect on their morphology, growth, or the amount of dead cells (**Figures 1A,C,D**). In turn, 24 h of Cd stress resulted in roots browning and inhibition of their growth (**Figures 1B,C**). Exposure for 24 h to higher Cd concentration led to significant increase in the amount of dead cells (**Figure 1D**).

**FIGURE 2:**
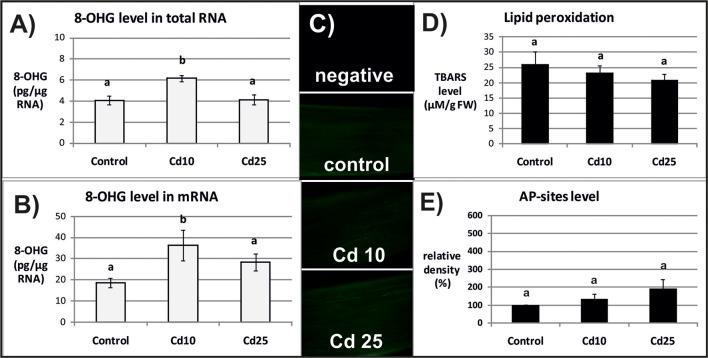
The level of 8-OHG in the total RNA **(A)**, mRNA **(B)**, general ROS level **(C)**, lipid peroxidation expressed as changes in TBARS level **(D)**, and the level of abasic (apurinic/apyrimidinic) sites (AP-sites) in mRNA expressed as percentage of relative density **(E)** after 3 h of treatment with distilled water (control) or Cd at the concentration 10 mgl^-1^ (Cd 10) or 25 mgl^-1^ (Cd 25). The results are means of 3–5 independent repetitions ± SE. Results marked with the same letter show no statistically significant differences.

**FIGURE 3:**
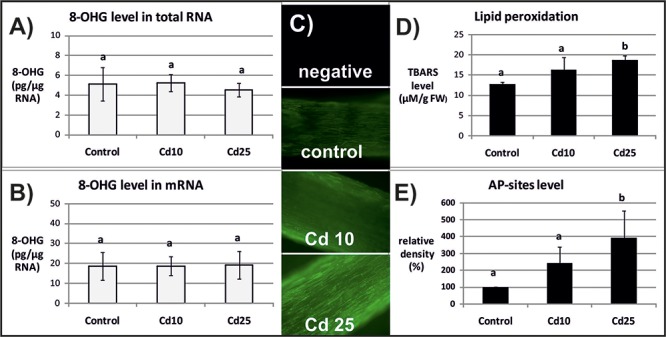
The level of 8-OHG in the total RNA **(A)**, mRNA **(B)**, general ROS level **(C)**, lipid peroxidation expressed as changes in TBARS level **(D)**, and the level of abasic (apurinic/apyrimidinic) sites (AP-sites) in mRNA expressed as percentage of relative density **(E)** after 24 h of treatment with distilled water (control) or Cd at the concentration 10 mgl^-1^ (Cd 10) or 25 mgl^-1^ (Cd 25). The results are means of 3–5 independent repetitions ± SE. Results marked with the same letter show no statistically significant differences.

The level of 8-OHG (oxidatively modified base) was approximately 5 times higher in mRNA in relation to the total RNA (**Figures 2A,B**, **3A,B**). In response to Cd, significant increase in the level of 8-OHG has been noted in the total RNA and mRNA after 3 h of treatment with lower concentration (10 mgl^-1^) (**Figures 2A,B**). This effect was not observed after 24 h or in response to higher Cd concentration (25 mgl^-1^) (**Figures 3A,B**).

The opposite tendency has been noted in the case of ROS accumulation. General ROS were detected with specific dye, CM-H_2_DCFD, which in response to oxidation emits green fluorescent signal. The fluorescence signal was generally lower after 3 than after 24 h. No differences in fluorescence intensity have been observed between the roots of control and Cd-stressed seedlings after 3 h (**Figure 2C**). However, a visible increase in the fluorescence signal has been noted in roots of the seedling treated with Cd for 24 h in relation to the control (**Figure 3C**).

The ROS over-production was correlated in time with increased lipid peroxidation (**Figure 3D**) and AP-sites frequency in the mRNA (**Figure 3E** and **Supplementary Figure S1**). After 3 h, no significant changes in TBARS or AP-sites level were observed between Cd-stressed and control seedlings (**Figures 2D,E**). In turn, after 24 h, the level of TBARS and AP-sites was significantly higher in the roots of seedlings treated with Cd at higher concentration (25 mgl^-1^) (**Figures 3D,E** and **Supplementary Figure S1**).

## Discussion

Exposure of plants to Cd leads to the development of various symptoms of toxicity (reviewed in Gallego et al., 2012). In the present study, 3 h long treatment with this metal did not affect the morphology and growth of soybean seedlings roots. At this time point, no statistically significant differences in cells viability were noted (**Figures 1A,C,D**). However, already after 24 h, browning and shortening of the roots in response to Cd treatment has been observed (**Figures 1A,C**). Additionally, 24 h long exposure to higher Cd concentration led to significant increase in the amount of dead cells (**Figure 1D**).

Cadmium (Cd) toxicity might be, at least partially, mediated by ROS. These molecules, which include hydrogen peroxide (H_2_O_2_), hydroxyl radical (HO), superoxide anion (O_2_^-^), and singlet oxygen (^1^O_2_), are highly reactive and mediate oxidation of various cellular compounds. Numerous reports showed that exposure to stresses leads to increase in the level of ROS accompanied by oxidation of proteins and membrane lipids (Gill and Tuteja, 2010; Cuypers et al., 2016; Sewelam et al., 2016). In fact, accumulation of the product of lipid peroxidation, malondialdehyde (MDA) and TBARS, is considered a typical symptom of oxidative stress. However, so far little attention has been given to the oxidation of nucleic acids in plant exposed to unfavorable condition. Moreover, the studies were limited only to changes in the level of DNA oxidation markers (Macovei et al., 2010; Yin et al., 2010), while there is no information concerning ROS impact on RNA. It is worth highlighting that studies on bacteria showed higher susceptibility of RNA to oxidation when compared to DNA. Under the same conditions, the level of oxidatively modified RNA exceeded level of oxidized DNA (Liu et al., 2012). This might be explained by the fact that DNA molecules are more protected due to localization in the nucleus, higher level of packing, and association with numerous proteins. Among the main RNA types, mRNA seems to be the most vulnerable to the oxidation (Bazin et al., 2011).

Indeed in the present study, the frequency of the most common oxidative modification, 8-OHG, was approximately 5 times higher in mRNA than in the total RNA (**Figures 2**, **3**). Another modification associated with oxidation processes, abasic sites (AP-sites), were undetectable in the total RNA using same or even higher concentrations of sample as in the case of mRNA (data not shown). The results indicate that also this modification occurs more frequently in the transcripts than other RNA types. Interestingly, Cd-dependent induction of 8-OHG and AP-sites were separated in time. Significantly, higher levels of 8-OHG in response to Cd were noted only after 3 h of treatment with lower metal concentration (**Figures 2A,B**). In turn, increase in the level of AP-sites was observed after 24 h of exposure to the higher concentration (**Figure 3E**) and was accompanied by increase in the level of other markers of oxidative stress – strong ROS over-production (**Figure 3C**) and enhanced lipid peroxidation (**Figure 3D**).

The observed variable in time effect of Cd-dependent ROS signal is in concordance with other studies. For example, several reports indicate that Cd stress leads to the generation of differing in the time of occurrence ROS waves (Garnier et al., 2006; Peréz-Chaca et al., 2014; Lv et al., 2017). In the case of the study on tobacco suspension cells, it has been shown that exposure to this metal leads to generation of three ROS waves, whereas the earliest one occurred already within the first hour of metal treatment and was dependent on the activity of membrane bond enzyme, NADPH oxidase. Same research reported that Cd cytotoxicity was associated with the second ROS wave resulting from disturbances in mitochondria functioning (Garnier et al., 2006). Also our earlier research showed that ROS signal in soybean seedlings exposed to Cd differs in time. The activity of NADPH oxidase modulated expression of signaling associated genes after 3 h of metal treatment, while significant ROS accumulation and lipid peroxidation were noted only after 24 h (Chmielowska-BąK et al., 2017). Apparently, the role of ROS in plant cells is temporal, species, and spatial specific (Miller et al., 2008; Cuypers et al., 2016). For example, it has been evidenced in *Arabidopsis thaliana* that ROS signal originating from peroxisomes and chloroplasts has distinct effect on the transcriptome (Sewelam et al., 2014). Another study using agents, which induce distinct ROS types, showed that some transcripts are modulated specifically by O_2_^-^, H_2_O_2_, or ^1^O_2_ (Gadjev et al., 2006).

Reactive oxygen species might play various roles in plants exposed to stress conditions. On one hand, these molecules are responsible for oxidative damage of membrane lipids, proteins, and nucleic acids (Das and Roychoudhury, 2014; Rio, 2015). On the other hand, ROS are engaged in signaling network and defense mechanism (Cuypers et al., 2016). ROS-dependent signaling includes direct sensing, for example, through transcription factors or serine/threonine protein kinase oxidative signal-inducible 1 (OXI1) and indirect modulation of signal through changes in cellular redox status and/or interaction with other signaling molecules such as nitric oxide, calcium ions, mitogen-activated protein kinases, or plant hormones (Neill et al., 2002; Chi et al., 2013; Kopczewski and Kuźniak, 2013; Wrzaczek et al., 2013; Waszczak et al., 2015; Cuypers et al., 2016). Recently, it has been proposed that ROS signal might be also transmitted by products of oxidation such as oxylipins, peptides derived from protein oxidation, and oxidatively modifies nucleic acids (reviewed in Chmielowska-BąK et al., 2015). One of the important future challenges in research concerning the role of ROS in plants response to stresses is elucidation of the exact role of specific ROS signals.

In the case of present research, the observed Cd-dependent induction of AP sites is most probably a symptom of oxidative stress (**Figure 3E** and **Supplementary Figure S1**). The assumption is based on the fact that AP sites induction was correlated in time with significant increase in ROS level and lipid peroxdiation (**Figures 3C,D**). However, at the present stage of research, it is difficult to explain the exact role of rapid induction of 8-OHG formation in RNA noted already after 3 h (**Figures 2A,B**), when the symptoms of oxidative stress were still not detectable (**Figures 2C,D**). Studies carried out on animal, human, and plant models showed that 8-OHG formation is not a random but highly selective process limited to defined transcripts, although the mechanism of its selectivity has not been yet discovered. High rate of 8-OHG in transcripts leads to ribosome stalling and in consequence to the decrease in the amount of encoded proteins (Shan et al., 2007; Tanaka et al., 2007; Chang et al., 2008; Bazin et al., 2011; Gao et al., 2013; Simms et al., 2014). The selective nature of 8-OHG formation and its impact on protein biosynthesis indicate that this process constitutes a newly discovered mechanism of post-transcriptional gene regulation. Interestingly, it seems that the gene regulatory function of transcripts abundant in 8-OHG plays distinct role in animals and plants. In the case of animal and human models, the high 8-OHG level in mRNA has been shown to be associated with the development of neurodegenerative disorders (Shan et al., 2003; Chang et al., 2008; Kong and Lin, 2010). In turn in plants, this oxidative modification of transcripts is essential for regulation of the level of certain proteins and alleviation of seed dormancy – a natural process in plants’ life cycle (Bazin et al., 2011; Gao et al., 2013).

In summary, this is the first report showing increased oxidation of total RNA and mRNA in plants exposed to stress. The observed increase in the level of AP-site was correlated in time with strong ROS accumulation and lipid peroxidation indicating that this mRNA modification constitutes a marker of oxidative challenge. However, the rapid induction of 8-OHG is puzzling and its exact role in plants response to unfavorable conditions needs further elucidation.

## Author Contributions

JC-B and JD designed the research. JC-B and AE-G carried out the cultivation of material, collection of samples, and RNA and mRNA isolation. JC-B and KI conducted the evaluation of ROS and AP-sites level. JC-B, MB, and AE-G performed the measurements of 8-OHG level including isolation of mRNA. JC-B carried out the examination of lipid peroxidation. All authors analyzed the obtained results. JC-B wrote the manuscript. All authors critically read, corrected, and approved the manuscript.

## Conflict of Interest Statement

The authors declare that the research was conducted in the absence of any commercial or financial relationships that could be construed as a potential conflict of interest.

## Supplementary Material

FIGURE S1 Photograph of an exemplary membrane showing formation of abasic sites (AP-sites) in mRNA isolated from the roots of seedlings treated with distilled water (control) or Cd at the concentration 10 mgl^-1^ (Cd 10) or 25 mgl^-1^ (Cd 25) for 3 and 24 h.

## Abbreviations

8-OHG: 8-hydroxyguanosine
AP-site: apurinic/apyrimidinic site
ARP: aldehyde reactive probe
CM-H_2_DCFDA 5-(and-6)-chloromethyl-2′,7′-dichlorodihydrofluorescein diacetate: acetyl ester
ROS: reactive oxygen species
TBARS: thiobarbituric acid reactive substances.
