# Supplementary figures and images for: Cadmium Stress Leads to Rapid Increase in RNA Oxidative Modifications in Soybean Seedlings
Source: Front Plant Sci. 2018 Jan 9;8:2219. doi: 10.3389/fpls.2017.02219 (PMC5767183; doi:10.3389/fpls.2017.02219)

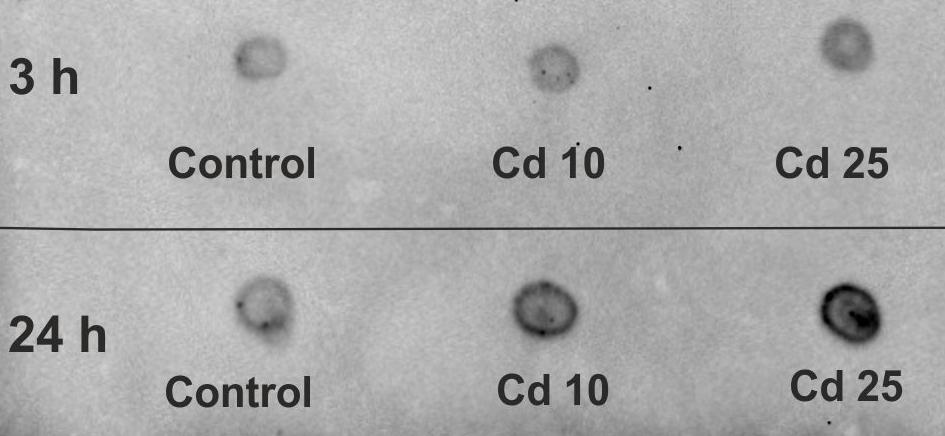

Supplement: FIGURE S1 — Photograph of an exemplary membrane showing formation of abasic sites (AP-sites) in mRNA isolated from the roots of seedlings treated with distilled water (control) or Cd at the concentration 10 mgl-1 (Cd 10) or 25 mgl-1 (Cd 25) for 3 and 24 h. [file Image_1.JPEG]
